# Acute Leukemia Presenting in the Pediatric Orbit

**DOI:** 10.7759/cureus.29996

**Published:** 2022-10-06

**Authors:** Pav Gounder, Siddharth Ogra, Kim Chan, Daisy Bassey-Duke, Yarrow Scantling-Birch, Linda Okafor, Raghavan Sampath, Simon N Madge

**Affiliations:** 1 Department of Ophthalmology, Sussex Eye Hospital, Brighton, GBR; 2 Department of Ophthalmology, Sir Charles Gairdner Hospital, Nedlands, AUS; 3 Department of Ophthalmology, Hereford County Hospital, Hereford, GBR; 4 Department of Ophthalmology, Counties Manukau District Health Board, Auckland, GBR; 5 Department of Ophthalmology, University Hospitals of Leicester, Leicester, GBR

**Keywords:** ocular symptoms, pediatrics ophthalmology, acute myeloid sarcoma, acute myeloid leukemia (aml), acute lymphoblastic leukemia (all)

## Abstract

We present a case series to evaluate the clinical features of acute leukemia presenting with primary orbital manifestations. We undertook a retrospective case review of primary orbital presentations of acute myeloid leukemia (AML) and acute lymphocytic leukemia (ALL) over a 10-year period at two hospital sites (Hereford County Hospital and Leicester Royal Infirmary). Our case series included four patients - two with AML and two with ALL. Patients were young (mean age of four years and five months) at presentation, all with unilateral disease, and presented with orbital signs. Although there was some confusion with the diagnosis at the time of referral, a suspicion of malignancy was made rapidly once ophthalmic review was initiated. All four cases were diagnosed with the assistance of peripheral blood film and bone marrow biopsy, without the need for orbital biopsy. All four cases had resolution of the orbital mass and remain disease-free.

## Introduction

Leukemia is the most common pediatric malignancy and is classified according to mode of presentation (acute or chronic) and cell line affected (lymphoid or myeloid) [1]. Orbital infiltration in leukemia is considered rare but can range between 14% and 66% and is associated with worse outcomes [2,3]. Orbital manifestations can act as an early window into systemic disease [4]. We report four cases of leukemias presenting in a pediatric population with orbital findings that were diagnosed using hematological and radiological findings and subsequently confirmed on bone marrow biopsy at a specialist unit. This case series adhered to the ethical principles outlined in the Declaration of Helsinki and written consent was obtained from all parents.

## Case presentation

Case 1

**Figure 1:**
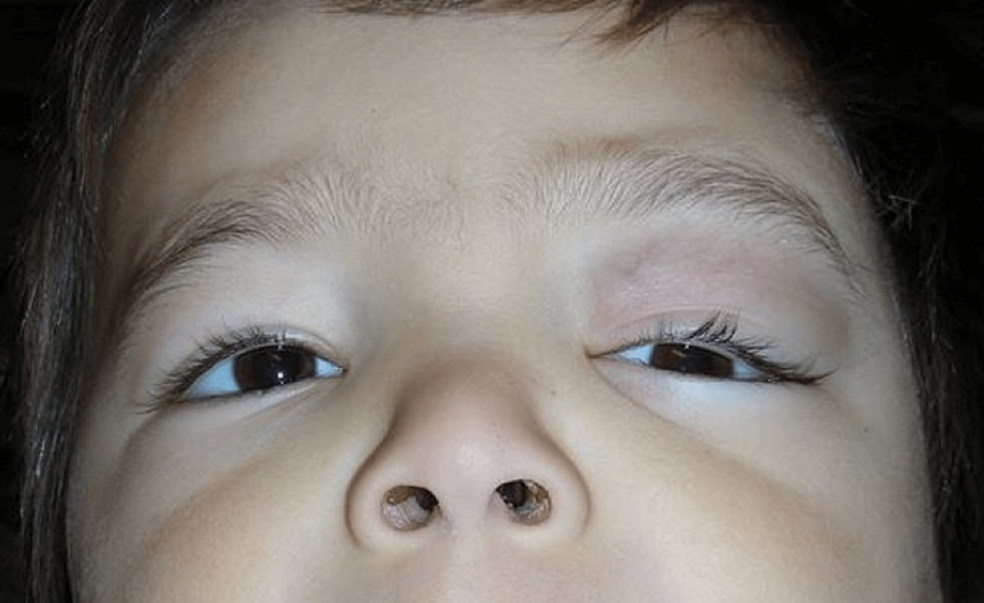
Clinical photo of a two-year and 10-month-old boy (case 1) presenting with left upper lid swelling, proptosis, and hypoglobus.

A boy aged two years and 10 months presented with a two-week history of left upper lid swelling with hypoglobus and proptosis, but no palpable orbital mass (Figure 1). Preauricular and cervical lymphadenopathy were present with possible hepatomegaly and splenomegaly.

**Figure 2:**
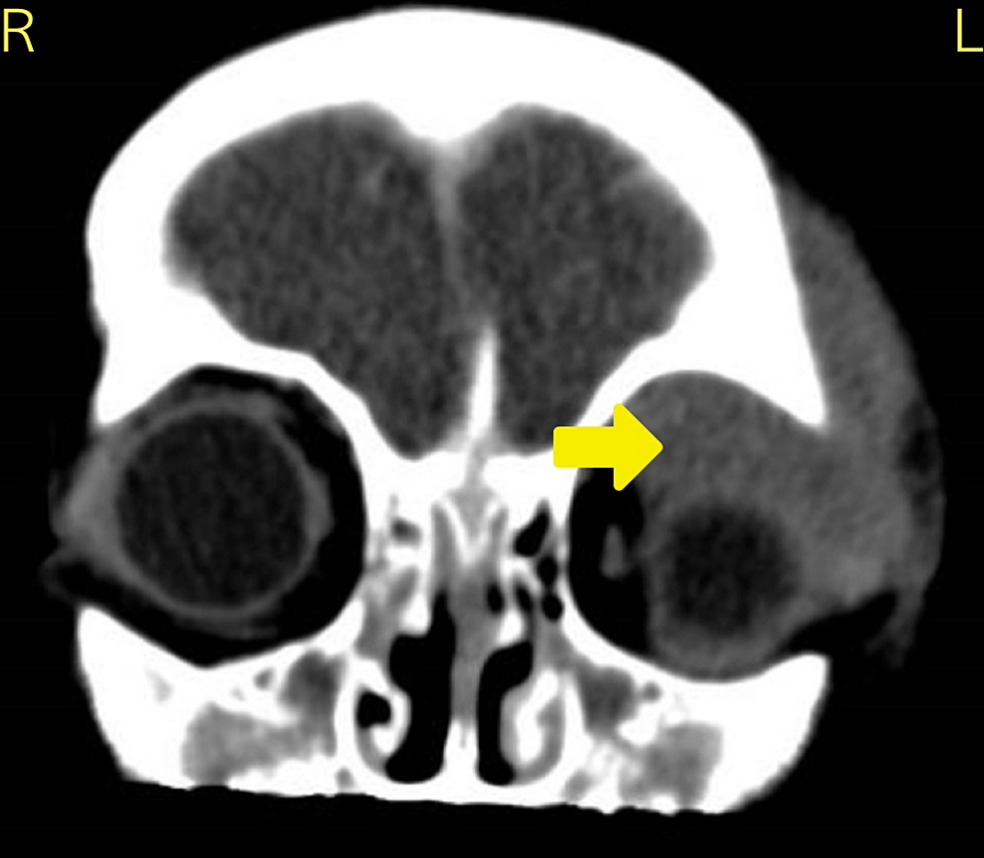
Coronal CT scan of brain and paranasal sinuses for case 1 revealed an aggressive left-sided superior orbital mass (arrow). There is inferior displacement of the globe, poor differentiation of superior and lateral recti muscles, and significant invasion of superotemporal soft tissue.

Circulating blasts were present in the peripheral blood film with a white cell count (WCC) of 16.6 x10^9^/L, red blood cells (RBC) of 1.45x10^12^/L, hemoglobin (Hb) of 40 g/L, platelets of 131x10^9^/L and neutrophil count of 1.99x10^9^/L (Table 1). There was a dimorphic RBC picture and smear cells were present. CT scan confirmed proptosis and a superior orbital mass (Figure 2).

**Table 1:** Peripheral blood film of a boy aged two years and 10 months (case 1) with acute lymphoid leukemia (ALL). Hb: hemoglobin; RBC: red blood cells; WCC: white cell count; NC: neutrophil count; PLT: platelet count

Laboratory test	Case 1 results	Reference ranges
Hb (g/L)	40	115-140
RBC (x10^12^/L)	1.45	3.9-5.3
WCC (x10^9^/L)	16.6	5.0-17.0
NC (x10^9^/L)	1.99	1.0-8.5
PLT (x10^9^/L)	131	150-400

The patient was referred to a pediatric specialist unit where he was diagnosed with acute lymphoid leukemia (ALL) with overexpression of cytokine receptor-like factor two following a bone marrow biopsy and started on the UKALL 2003 chemotherapy regimen C (vincristine, dexamethasone, PEG asparaginase, doxorubicin, cyclophosphamide, cytarabine, and methotrexate). He remains in remission five years post-cessation of chemotherapy with no recurrence of eye symptoms and resolution of orbital mass.

Case 2

**Figure 3:**
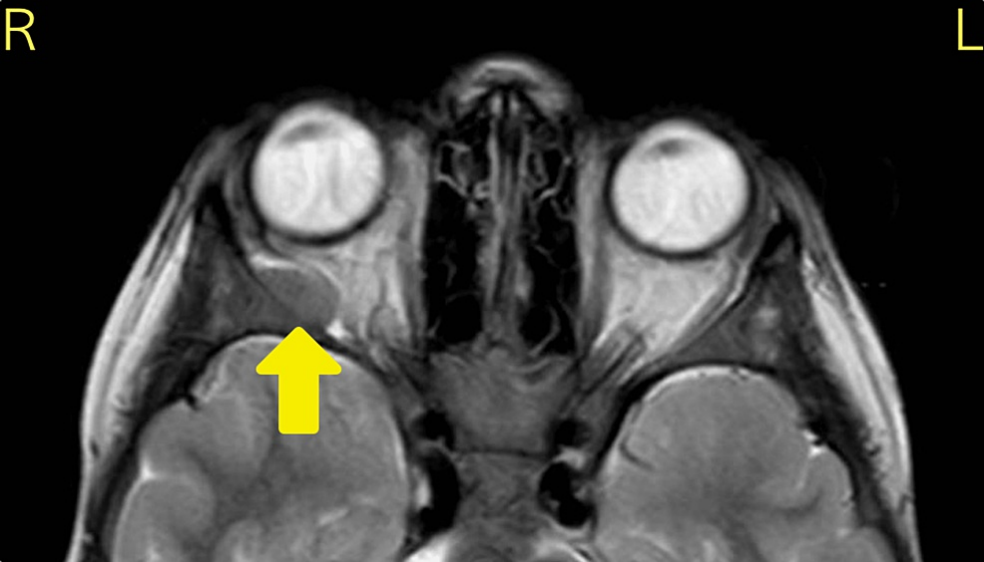
Axial T2 MRI head and orbits for case 2 demonstrated a well-defined, hypointense 12.7x7 mm extraconal mass in the lateral wall of the right orbit (arrow). There is displacement of the right lateral rectus muscle, but no local invasion of other orbital structures.

A seven-year-old girl presented with injected conjunctiva with an associated lesion on the nasal conjunctiva of the right eye on a background of left knee pain of four days duration. She had experienced right eye pain that was initially diagnosed as episcleritis. She returned three days later with ptosis and diplopia. Ocular examination demonstrated an abduction deficit. MRI of the head and orbits demonstrated a 12.7x7 mm mass in the lateral wall of the right orbit that displaced the lateral rectus muscle (Figure 3). Her peripheral blood film revealed a large population of circulating blasts and Auer rods. She had a WCC of 7x10^9^/L, RBC of 2.1x10^12^/L, Hb of 71 g/L, platelets of 35x10^9^/L, and neutrophils of 1.05x10^9^/L (Table 2).

**Table 2:** Peripheral blood film for a seven-year-old girl (case 2) with acute myeloid leukemia (AML). Hb: hemoglobin; RBC: red blood cells; WCC: white cell count; NC: neutrophil count; PLT: platelet count

Laboratory test	Case 2 results	Reference ranges
Hb (g/L)	71	115-155
RBC (x10^12^/L)	2.1	4.0-5.2
WCC (x10^9^/L)	7.0	4.5-14.5
NC (x10^9^/L)	1.05	1.0-8.0
PLT (x10^9^/L)	35	150-400

She was referred to a pediatric specialist unit where she was diagnosed, following bone marrow biopsy, with CNS-positive acute myeloid leukemia (AML) with RUNX1-RUNX1T1 fusion and commenced on treatment (mitoxantrone, high dose cytarabine, and triple intrathecal therapy). She remains in remission four years post-cessation of chemotherapy with no recurrence of periocular symptoms and radiographic evidence of mass resolution.

Case 3

A boy aged four years and 10 months who had a history of left esotropia from the age of two years was noted to have swelling on the nasal aspect of his left lower lid during routine orthoptist assessment, which was present for a two-month period. He was initially treated for left dacryocystitis, but one week later presented to eye casualty following an increase in swelling of the left lower lid.

**Figure 4:**
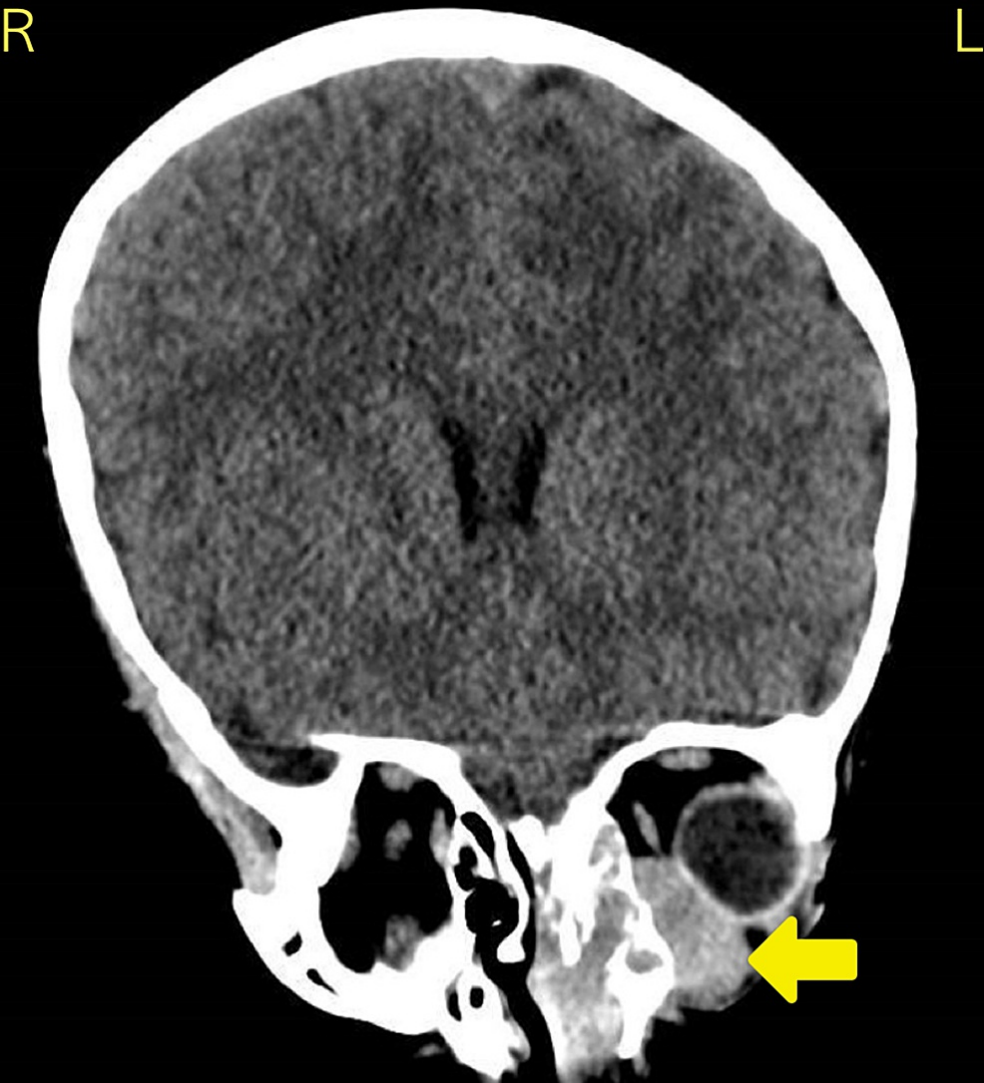
Coronal CT brain and paranasal sinuses for case 3 revealed a left-sided orbital mass (arrow) occupying the inferonasal aspect of the orbit. There is local invasion through the lamina papyracea of the left ethmoid air cells, nasal cavity, and mild displacement of the nasal septum.

CT of the brain and paranasal sinuses revealed an orbital mass occupying the inferonasal aspect of the orbit that extended into the nasal cavity (Figure 4). WCC was 4.2x10^9^/L, RBC was 3.44x10^12^/L, Hb was 40 g/L, platelets were 131x10^9^/L, and neutrophils were 1.99x10^9^/L (Table 3).

**Table 3:** Peripheral blood film of boy aged four years and 10 months (case 3) with acute myeloid leukemia (AML). Hb: hemoglobin; RBC: red blood cells; WCC: white cell count; NC: neutrophil count; PLT: platelet count

Laboratory test	Case 3 results	Reference ranges
Hb (g/L)	40	115-140
RBC (x10^12^/L)	3.44	3.9-5.3
WCC (x10^9^/L)	4.2	5.0-17.0
NC (x10^9^/L)	1.99	1.0-8.5
PLT (x10^9^/L)	131	150-400

Care was transferred to a specialist pediatric unit and the patient, following bone marrow biopsy, was diagnosed with AML with RUNX1-RUNX1T1 fusion for which he was started on the MyeCHILD chemotherapy regimen (mitoxantrone, cytarabine, and gemtuzumab ozogamicin). He had a negative PET-CT scan after two cycles and negative flow cytometry minimal residual disease post-course one. He remains in complete remission three years post-diagnosis with resolution of the orbital mass.

Case 4

A three-year-old boy presented with a two-day history of right peri-orbital swelling as well as right leg pain. Clinical examination revealed significant right upper lid swelling with pseudoptosis and mild proptosis but no palpable mass. The patient was referred to the specialist orbital team and a second review of the images highlighted abnormal bone texture in addition to sinus disease in the body of the sphenoid and abnormal bone texture in the angle of the mandibles. Peripheral blood film demonstrated circulating blasts. WCC was 29.4x10^9^/L, RBC was 4.97x10^12^/L, Hb 125 g/L, and platelets were 319x10^9^/L (Table 4).

**Table 4:** Peripheral blood film of a three-year-old boy (case 4) with acute lymphoid leukemia (ALL). Hb: hemoglobin; RBC: red blood cells; WCC: white cell count; PLT: platelet count

Laboratory test	Case 4 results	Reference ranges
Hb (g/L)	125	115-140
RBC (x10^12^/L)	4.97	3.9-5.3
WCC (x10^9^/L)	29.4	5.0-17.0
PLT (x10^9^/L)	319	150-400

He was transferred to the pediatric unit with a formal diagnosis of ALL with ETV6-RUNX1 fusion gene and commenced on the UKALL 2011 chemotherapy regimen A (vincristine, dexamethasone, PEG asparaginase, doxorubicin, cyclophosphamide, and cytarabine). There was complete resolution of his peri-orbital swelling within five days of starting chemotherapy, and he remains in remission 18 months post-diagnosis.

## Discussion

Leukemia can present with ocular findings after systemic diagnosis, as the first presenting sign, or as the manifestation of relapsing disease [3,5]. Orbital involvement is more frequently described in cases of AML [6], but both acute leukemias have similar clinical manifestations if infiltrating the orbit [7]. Achieving an early diagnosis is critical as ocular manifestations of acute leukemias have previously been associated with worse systemic disease or higher risk of relapse [3,8].

The laterality of pediatric orbital lesions can help tailor a differential diagnosis. Rhabdomyosarcoma, secondary tumors, and cystic lesions are typically unilateral. The most common bilateral lesions tend to be inflammatory, related to orbital metastasis of neuroblastoma, or myeloid sarcoma [9]. In 1986, Shields et al. analyzed a series of case reports of orbital myeloid sarcoma and 60% (n=53) presented with bilateral lesions [10]. However, we present two cases of AML and two ALL with unilateral presentations. Given the demographic of the case series by Shields et al. was older, we hypothesize that with improved diagnostic modalities and access to healthcare, patients are presenting earlier and possibly prior to bilateral orbital manifestations [10].

Orbital biopsies prove to be useful diagnostic procedures in identifying the etiology of space-occupying orbital lesions. In cases of acute leukemia, an orbital biopsy may be required if a diagnosis cannot be made confidently on the grounds of clinical and radiological findings, or when orbital findings are the first presenting complaint. Orbital incisional biopsies carry procedural risks in a complex anatomical space. Core-needle and fine-needle aspiration biopsies have gained popularity as minimally invasive techniques, but in themselves carry the risk of inadequate tissue yield. Our case series demonstrates the synergistic use of clinical and hematological findings to raise the suspicion of acute leukemia and referral to specialist pediatric unit. Non-invasive and inexpensive investigations, such as a full blood count and peripheral blood smear, should be considered first in a work-up before surgical biopsy in the diagnosis of acute leukemia with orbital involvement. In pediatric case series of orbital leukemia, the patients that required an orbital biopsy for definitive diagnosis remain in the minority, ranging from 7.4% to 17% [6,11].

## Conclusions

Although leukemia is the most common childhood malignancy, orbital presentations remain rare but should not be forgotten given the potential consequences. Orbital lesions of ALL are less common than AML but also require prompt diagnosis and treatment. There is generally good survival for both entities, however, orbital manifestations may be associated with worse outcomes. Diagnosis can be achieved without the need for orbital biopsy using hematological and radiological findings. Multidisciplinary collaboration between ophthalmology, hematology, oncology, and radiology is essential.

## References

[1] Steliarova-Foucher E, Colombet M, Ries LA (2017). International incidence of childhood cancer, 2001-10: a population-based registry study. Lancet Oncol.

[2] Kincaid MC, Green WR (1983). Ocular and orbital involvement in leukemia. Surv Ophthalmol.

[3] Russo V, Scott IU, Querques G, Stella A, Barone A, Noci ND (2008). Orbital and ocular manifestations of acute childhood leukemia: clinical and statistical analysis of 180 patients. Eur J Ophthalmol.

[4] Chaudhry SR, Kreis AJ, Underhill HC, Madge SN (2014). Orbital mass secondary to acute lymphoblastic leukaemia in a child: a rare presentation. Orbit.

[5] Sharma T, Grewal J, Gupta S, Murray PI (2004). Ophthalmic manifestations of acute leukaemias: the ophthalmologist's role. Eye (Lond).

[6] Bidar M, Wilson MW, Laquis SJ (2007). Clinical and imaging characteristics of orbital leukemic tumors. Ophthalmic Plast Reconstr Surg.

[7] Thakker MM, Rubin PA, Chang E (2006). Pre-B-cell acute lymphoblastic leukemia presenting as an orbital mass in an 8-month-old. Ophthalmology.

[8] Zimmerman LE, Font RL (1975). Ophthalmologic manifestations of granulocytic sarcoma (myeloid sarcoma or chloroma). Am J Ophthalmol.

[9] Shields JA, Stopyra GA, Marr BP, Shields CL, Pan W, Eagle RC Jr, Bernstein J (2003). Bilateral orbital myeloid sarcoma as initial sign of acute myeloid leukemia: case report and review of the literature. Arch Ophthalmol.

[10] Shields JA, Bakewell B, Augsburger JJ, Donoso LA, Bernardino V (1986). Space-occupying orbital masses in children. A review of 250 consecutive biopsies. Ophthalmology.

[11] Murthy R, Vemuganti GK, Honavar SG, Naik M, Reddy V (2009). Extramedullary leukemia in children presenting with proptosis. J Hematol Oncol.

